# Comparison between the STENTYS self-apposing bare metal and paclitaxel-eluting coronary stents for the treatment of saphenous vein grafts (ADEPT trial)

**DOI:** 10.1007/s12471-017-1066-0

**Published:** 2017-12-18

**Authors:** A. J. J. IJsselmuiden, C. Simsek, A. G. van Driel, D. Bouchez, G. Amoroso, P. Vermeersch, P. P. Karjalainen

**Affiliations:** 1 Department of Cardiology, Albert Schweitzer Hospital, Dordrecht, The Netherlands; 2 Department of Cardiology, Erasmus Medical Centre, Rotterdam, The Netherlands; 3 STENTYS, STENTYS SA, Paris, France; 4 Department of Cardiology, Onze Lieve Vrouwe Gasthuis, Amsterdam, The Netherlands; 5 Department of Cardiology, ZNA Middelheim, Antwerpen, Belgium; 6 Department of Cardiology, Satakunta Hospital, Pori, Finland

**Keywords:** Percutaneous coronary intervention, Drug-eluting stent, Bare metal stent, Self-apposing stent, Saphenous vein grafts

## Abstract

**Aims:**

To describe the safety and performance of STENTYS self-expandable bare metal stents (BMS) versus paclitaxel-eluting stents (PES) in saphenous vein grafts (SVGs).

**Methods and Results:**

A randomised controlled trial was performed in four hospitals in three European countries between December 2011 and December 2013. Patients with *de novo* lesions (>50% stenosis) in an SVG with a diameter between 2.5–6 mm were included. Primary endpoint was late lumen loss at 6 months. Secondary endpoints included procedural success and the occurrence of major adverse cardiac events (MACE) at 12 months. A total of 57 patients were randomised to STENTYS self-apposing BMS (*n* = 27) or PES (*n* = 30). Procedural success was obtained in 89.5%. No significant differences in late lumen loss were found between BMS and PES at 6 months (0.53 mm vs 0.47; *p* = 0.86). MACE rates at 12 months were comparable in both groups (BMS 22.2% vs. PES 26.7%; *p* = 0.70).

**Conclusions:**

Treatment of SVGs with STENTYS self-expandable stents is safe and effective. No significant differences were found in late lumen loss and MACE between BMS and PES.

## Introduction

Saphenous vein grafts (SVGs) can occlude over time because of thrombosis, intimal hyperplasia and atherosclerosis. Occlusions of SVGs are reported in 10–15% of patients within 1 year after coronary artery bypass grafting (CABG) surgery and after 10 years almost half of the SVGs fail [1]. Currently 10% of the patients being treated in a high-volume catheterisation lab are patients who need treatment of their occluded SVGs [2]. These patients can encounter complications such as embolisation, peri-procedural myocardial infarction (MI), increased incidence of restenosis, repeat percutaneous coronary intervention (PCI) and a faster progression of moderate ‘non-significant’ lesions, treated during the first intervention [3, 4]. Originally SVGs were treated with balloon angioplasty alone. Subsequently the SAVED trial showed beneficial procedural results and a decrease in major adverse cardiac events (MACE) with the use of bare metal stents (BMS) compared with balloon angioplasty [5].

Since the introduction of drug-eluting stents (DES) at least two meta-analyses have reported lower MACE and repeat revascularisation rates with DES but, overall, similar mortality rates [6, 7]. However, the majority of the articles in the meta-analyses were observational studies. Recently a meta-analysis with only four randomised controlled trials showed that the use of DES in SVGs was associated with a significant reduction in risk of repeat revascularisation. In addition, there were no differences in the incidence of all-cause death and nonfatal MI between DES and BMS [8].

Besides a lack of methodological evidence (randomised controlled trials, RCTs) in the assessment of PCI treatment of SVGs, there is also limited information about the role of self-expandable stents. The implantation of self-expandable stents in SVGs has theoretical advantages compared with balloon-expandable stents, such as less risk of undersizing or oversizing in a vessel with a large lumen diameter and calibre change, especially at the anastomosis site. Secondly, deployment could limit the risk of distal embolisation as it is deployed in a distal to proximal direction. Third, the self-expanding mechanism could improve apposition after thrombus resolution following PCI. The Symbiot III study, a randomised trial with 400 patients, compared BMS with a self-expanding polytetrafluoroethylene covered stent in the treatment of *de novo *and restenotic SVG lesions. No differences were reported in the MACE rate at 8 months or in angiographic restenosis [9]. As discussed before, the optimal treatment (BMS or DES) in SVGs has yet not been established.

For this reason, a randomised study, systematically comparing the safety and efficacy of a self-expanding BMS with a self-expanding DES in SVGs, may provide meaningful results and lead to improved treatment of SVGs.

## Methods

### Patient population

Patients were eligible if they were at least 18 years of age and were willing to comply with the follow-up evaluations. Angiographic eligibility criteria were *de novo* lesions (>50% stenosis) in SVGs with a diameter between 2.5–6 mm by visual estimation. Finally, patients had to understand the nature of the procedure and provide written informed consent prior to the procedure. The following exclusion criteria were applied: patients with cardiogenic shock, any vasculature lesions or characteristics preventing the safe performance of a PCI with the STENTYS delivery system or placement of the STENTYS stent, allergies or contraindications to antiplatelet medication, known allergies to stent components and female patients with child bearing potential not taking adequate contraceptives or currently breastfeeding.

Study personnel approached the patients who met the inclusion criteria. Patients were told that even if they were willing to participate in the study, further examinations might demonstrate that they were not suitable. Written informed consent was obtained from all patients. The study complied with the Declaration of Helsinki and was approved by the Ethics Committees of the sites before study enrolment commenced.

### Study design and procedures

**Fig. 1:**
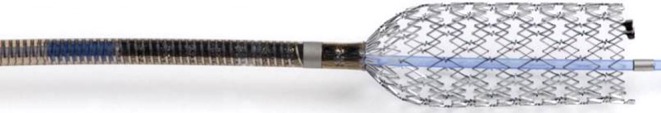
The STENTYS stent is deployed by retracting an outer sheath which releases the stent from the distal edge

Patients were included at four medical centres in Europe (Albert Schweitzer Hospital, Dordrecht, the Netherlands; Onze Lieve Vrouwe Gasthuis, Amsterdam, the Netherlands; ZNA Middelheim, Antwerpen, Belgium; and Satakunta Hospital, Pori, Finland). Eligible patients were consecutively randomised to treatment with a STENTYS BMS or a STENTYS paclitaxel-eluting stent (PES). The allocation schedule was based on computer-generated random numbers on a 1:1 basis. The study was conducted on the STENTYS self-expanding PES or BMS, which feature small interconnections that can be disconnected by inflating a balloon catheter to provide access to the side branch and full ostium coverage[10]. The concept of the device is shown in Fig. 1. Self-expanding stents have been proven to be safe and effective in the treatment of patients with coronary bifurcation lesions[11] and STEMI patients[12]. Both stents were self-expanding with a nickel titanium alloy (nitinol) on a rapid exchange delivery system. The stents were available in diameters of 2.5–4.5 mm and in lengths of 17–27 mm. Balloon angioplasty and coronary stent implantation were performed using standard techniques. The use of a filter-wire or any other distal protection was encouraged but the final decision was at the operator’s discretion. Pre- and post-dilatation were recommended, although left to the discretion of the operator. All patients were treated with at least 80 mg acetylsalicylic acid and 300–600 mg clopidogrel before or at the time of the procedure. During PCI, unfractionated heparin in a dose of at least 100 IU/kg was administered to maintain an activated clotting time >200 s. The use of glycoprotein IIb/IIIa antagonists was left to the discretion of the operator. All patients were asked to return for angiographic follow-up at 6 months (±30 days).

### Quantitative coronary angiography

Coronary angiograms were digitally recorded at baseline and at 6 months (±30 days) follow-up. An independent core lab assessed the images (Diagram BV, Zwolle, the Netherlands). The projections that best showed the stenosis were used for analysis. Quantitative measurements included the diameter of the vessel, the minimal luminal diameter (MLD), percent diameter stenosis, and late lumen loss which was defined as the difference between MLD after the procedure and MLD at follow-up. Binary stenosis was defined as stenosis of 50% or greater of the MLD in the target lesion at angiographic follow-up. All angiographic measurements of the target lesion were obtained in the stented area, within the margins 5 mm proximal and distal to each stent edge.

### Cardiac clinical outcomes

The primary endpoint of this study was in-stent late lumen loss at 6‑month follow-up, assessed by quantitative coronary angiography (QCA). The main secondary endpoints were: procedural success (defined as attainment of <30% final residual stenosis and no periprocedural complications), major adverse cardiac events at 12 months (MACE; defined as cardiac death, MI, emergent CABG or clinically driven target lesion revascularisation), target vessel failure (TVF; defined as cardiac death, target vessel myocardial infarction or clinically driven target vessel revascularisation) at 12 months, binary restenosis at 6 months and stent strut malapposition at 6 months.

### Sample size

The primary measure of patient outcome was in-stent late lumen loss at 6‑month follow-up. To show a difference between the mean per-lesion in-stent late lumen loss in the STENTYS BMS group (estimated lumen loss: 1.0 ± 0.9 mm) and the STENTYS PES group (estimated lumen loss: 0.5 ± 0.7 mm), 40 analysable patients needed to be enrolled in both groups, assuming a 1:1 patient allocation ratio of the treatments and a type I error (α) of 0.05 (two-sided). However, because enrolment was slower than anticipated, 57 patients were randomised (27 patients to the STENTYS BMS stent and 30 patients to the STENTYS PES stent).

### Statistical analysis

The primary analysis sample was based on the principle of intention-to-treat using SPSS (SPSS Inc., Chicago, Illinois). In the intention-to-treat population, all patients who gave informed consent and were randomised were included in the analysis sample, regardless of whether or not the treatment device was successfully implanted. The per-protocol population were the population who fulfilled the inclusion and exclusion criteria, who received the treatment device they were randomised to and who completed the 6‑month follow-up. The primary endpoint is the in-stent late lumen loss at 6 months of follow-up; means and standard deviations per treatment group were reported. Student’s t‑test for independent groups was used to test for statistical significance of the primary endpoint. The test is two-sided and an alpha of 5% was used as the level of significance. Levene’s test for equality of variances was conducted and if necessary, a modified t‑test that does not assume equality of variance was used. We checked for normality of in-stent late lumen loss scores by means of visual examination. If necessary these scores were transformed to obtain a normal distribution. For secondary outcomes, standard Chi-square tests was performed to compare percentages of categorical data. In case of rare events (the expected number per cell lower than 5 in more than 20% of the cells) the Fisher’s exact test was used. For ordinal categorical data a chi-square test for linear trend was performed. Depending on the distribution of the data, T‑tests or Mann-Whitney U tests were used for continuous data. All tests are two-sided and an alpha of 5% was used as the level of significance.

## Results

### Baseline and procedural characteristics

**Fig. 2:**
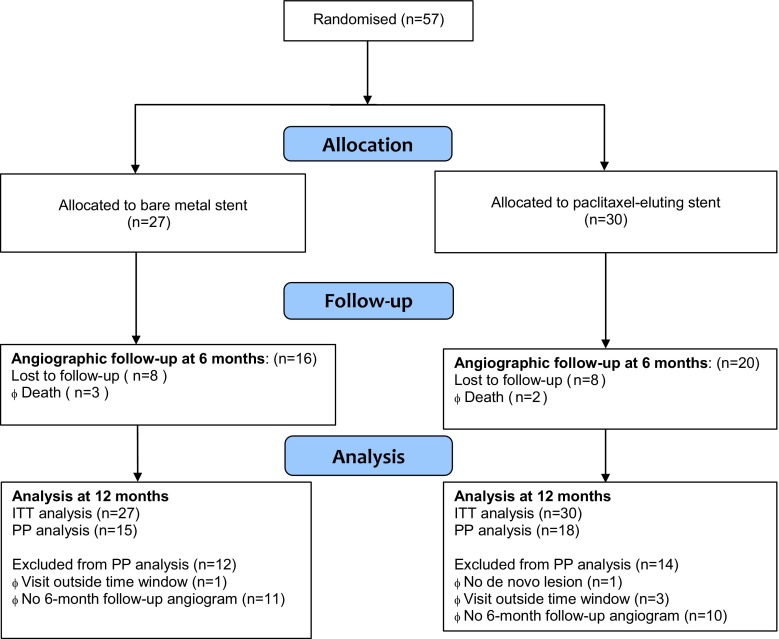
Study flow chart. Allocation, follow-up and analysis. Lost to follow-up: if the patient missed two consecutive scheduled contact time points and attempts at communicating with the patient were unsuccessful, the patient was considered lost-to-follow-up. (*ITT analysis*  Intention-to-treat analysis; all patients who gave informed consent and were randomised, *PP analysis* Per-protocol analysis; the patients who fulfil the inclusion and exclusion criteria, who have received the treatment device they were randomised to and who have completed the 6‑month follow-up)

Between December 2011 and December 2013*, *57 patients were consecutively enrolled in the ADEPT trial and were randomised to BMS (*n* = 27) or PES (*n* = 30) (Fig. 2). Baseline and procedural characteristics were not statistically different in both groups (Tab. 1).

**Table 1:** Baseline and procedural characteristics of the study population

	BMS (*n* = 27)	PES (*n* = 30)	All (*n* = 57)	*P* Value
Age (years)	73.3 ± 7.9	73.5 ± 9.8	73.4 ± 8.9	0.92
Male gender	25 (92.6%)	25 (83.3%)	50 (87.7%)	0.43
Age graft (years)	15.9 ± 8.7	16.5 ± 7.7	16.2 ± 8.1	0.77
*Cardiac risk factors*				
Diabetes mellitus	9 (33.3%)	10 (33.3%)	19 (33.3%)	0.99
Hypertension	15 (55.6%)	20 (66.7%)	35 (61.4%)	0.39
Hypercholesterolaemia	15 (55.6%)	19 (63.3%)	34 (59.6%)	0.55
Current smoking	3 (11.1%)	5 (16.7%)	8 (14.0%)	0.71
Family history of CAD	9 (33.3%)	14 (46.7%)	23 (40.4%)	0.59
*Past medical history*				
Myocardial infarction	12 (44.4%)	15 (50.0%)	27 (47.4%)	0.59
Previous PCI	13 (48.1%)	15 (50.0%)	28 (49.1%)	0.89
Previous CABG	27 (100.0%)	30 (100.0%)	57 (100%)	0.99
Previous stroke	0 (0%)	4 (13.3%)	4 (7.0%)	0.11
*Indication for PCI*				
Stable angina	11 (40.7%)	14 (46.7%)	25 (43.9%)	0.65
Unstable angina	12 (44.4%)	11 (36.7%)	23 (40.4%)	0.55
Silent ischaemia	1 (3.7%)	2 (6.7%)	3 (5.3%)	0.99
Other	3 (11.1%)	3 (10.1%)		0.99
*Number of stents used at target lesion*				
1	26 (96.3%)	30 (100%)	56 (98.2%)	0.47
2	6 (22.2%)	5 (16.7%)	11 (19.3%)	0.60
3	1 (3.7%)	2^a^ (6.7%)	3 (5.3%)	0.99
*QCA Pre-procedural results*				
Lesion length, mean (mm)	15.92 ± 7.34	16.92 ± 7.89	16.44 ± 7.57	0.59
Reference diameter (mm)	3.11 ± 0.84	3.26 ± 0.58	3.19 ± 0.71	0.96
Minimal lumen diameter (mm)	1.36 ± 0.64	1.34 ± 0.56	1.35 ± 0.59	0.92
Stenosis, % lumen diameter	55.02 ± 19.52	58.00 ± 17.98	56.56 ± 18.63	0.49
Embolic protection device use	4 (14.8%)	6 (20%)	10 (17.5%)	0.73
*Success rates*				
Device success	26 (96.3%)	28 (93.3%)	54 (94.7%)	0.99
Procedure success	25 (92.6%)	26 (86.7%)	51 (89.5%)	0.67
Clinical success	25 (92.6%)	26 (86.7%)	51 (89.5%)	0.67

Values are mean ± SD or *n* (%)

*BMS* bare-metal stent(s), *PES* paclitaxel-eluting stent(s), *CAD* coronary artery disease, *PCI* percutaneous coronary intervention, *CABG* coronary artery bypass graft, *SVG* saphenous vein grafts, *QCA* quantitative coronary angiography

^a^Different stent types were implanted (non-STENTYS stent)

Most of the treated patients were men (87.7%). The mean age of the population was 73.4 ± 8.9 years. Most PCI indications for SVG treatment were stable angina (43.9%) and unstable angina (40.4%). The mean graft age was 16.2 years. Embolic protection was used in 10 (17.5%) lesions; no distal embolisation was reported. Procedural success was achieved in 51 (89.5%) patients.

### Primary endpoints

Angiographic follow-up was completed according to protocol, 6 months after stent implantation, in 40 of the 57 patients (70.1%). The follow-up angiographic results are presented in Tab. 2. No significant differences in late lumen loss were found between the BMS and PES group: 0.53 mm ± 1.09 and 0.47 mm ± 0.95 respectively (*p* = 0.86).

**Table 2:** Lumen area development‡

	BMS (*n* = 27)	PES (*n* = 30)	All (*n* = 57)	*P* value
*Diameter stenosis (%)*				
Pre procedural	55.02 ± 19.52	58.00 ± 17.98	56.56 ± 18.63	0.49
Post procedural	8.08 ± 11.48	7.07 ± 13.89	7.54 ± 12.72	0.97
6 months FU	23.72 ± 32.30	21.25 ± 30.18	22.36 ± 30.77	0.84
*RVD (mm)*				
Pre procedural	3.11 ± 0.84	3.26 ± 0.58	3.19 ± 0.71	0.97
Post procedural	3.22 ± 0.51	3.17 ± 0.53	3.19 ± 0.52	0.97
6 months FU	3.30 ± 0.60	3.11 ± 0.53	3.19 ± 0.56	0.47
*MLD (mm)*				
Pre procedural	1.36 ± 0.64	1.34 ± 0.56	1.35 ± 0.59	0.92
Post procedural	2.93 ± 0.50	2.91 ± 0.47	2.92 ± 0.48	0.94
6 months FU	2.47 ± 1.07	2.43 ± 0.98	2.45 ± 1.01	>0.90
LLL after 6 months (mm)	0.53 ± 1.09	0.47 ± 0.95	0.50 ± 1.00	0.86
Binary restenosis at 6 months (>50%)	3/18 (16.7%)	2/22 (9.1%)	5/40 (12.5%)	0.64

*RVD* reference vessel diameter, *MLD* minimal lumen diameter,* LLL* late lumen loss

Values are mean ± SD or *n* (%). ‡ In 1 patient a QCA preanalysis was not possible because the catheter tip was not filmed

The numbers of patients returning for angiographic 6 months follow-up: BMS (*n* = 16), PES (*n* = 20), All (*n* = 36)

### Secondary endpoints

The clinical outcomes up to 1‑year of follow-up are presented in Tab. 3. During follow-up to 365 days, 14 of 57 patients experienced at least 1 MACE: 6 of 27 BMS patients and 8 of 30 PES patients (*p* = 0.70). There were also no significant differences in TVF rates between both groups (BMS group 22.1%; PES group 20.0%; *p* = 0.84). Five of the 57 patients died during follow-up: 3 patients in the BMS and 2 in the PES group. The 2 patients who died in the PES group were non-cardiac deaths (1 due to urosepsis and 1 due to urothelial cell carcinoma). The 3 patients in the BMS group were cardiac deaths (1 sudden death and 2 with unknown cardiac cause). Six of the 57 patients experienced an MI (1 in the BMS and 5 in PES group; *p* = 0.20) during follow-up. Target vessel revascularisation was reported in 3 of the 6 MI patients: 1 in the BMS group (NSTEMI) and 2 in the PES group (1 acute in-stent thrombosis within 30 days, and 1 in-stent restenosis and in-stent thrombosis within 180 days). The other 3 patients with MI were related to non-target vessels. Clinically driven target lesion revascularisation was necessary in 9 of the 57 patients (4 in BMS and 5 in PES; *p* = 0.99). Binary restenosis at 6 months was reported in 5 of 40 patients, including 3 in the BMS group and 2 in the PES group (*p* = 0.64).

**Table 3:** Clinical outcomes during follow-up

	BMS (*n* = 27)	PES (*n* = 30)	All (*n* = 57)	*P* Value
*MACE rate*				
In hospital MACE	0 (0%)	0 (0%)	0 (0%)	0.99
MACE within 30 days	1 (3.7%)	2 (6.7%)	3 (5.3)	0.99
MACE within 180 days	3 (11.1%)	5 (16.7%)	8 (14.0)	0.71
MACE within 365 days	6 (22.2%)	8 (26.6%)	14 (24.6)	0.70
*TVF rate*				
TVF within 365 days	6 (22.2%)	6 (20.0)	12 (21.1)	0.84
Death, any cause	3 (11.1%)	2 (6.7%)	5 (8.8%)	0.66
Cardiac Death	3 (11.1%)	0 (0%)	3 (5.7%)	0.10
MI, any	1 (3.7%)	5 (16.7%)	6 (10.5%)	0.20
Clinical driven TLR	4 (14.8%)	5 (16.7%)	9 (15.8%)	0.99

*MACE* major adverse cardiac events, *MI* myocardial infarction, *TLR* target lesion revascularisation

Values are mean ± SD or *n* (%)

## Discussion

STENTYS self-expandable stents have the unique feature of positive adaptation to the vessel size. When adrenergic activation leading to vasoconstriction diminishes in the days after PCI, and dual antiplatelet medication starts to dissolve the encaged thrombus, a stent self-expanding to an optimal size and apposition may have additional benefits [13].

Especially in SVGs, with a larger vessel size than a native coronary artery, the risk of undersizing is reduced by the STENTYS self-expandable stents. Furthermore, the potential for distal embolisation could be reduced as the stent deploys in a distal to proximal direction; in the current study no distal embolisation was reported. Finally, vessel wall injury, which has a direct relation with neo-intimal proliferation, might be limited by self-expanding stents compared with balloon-expanding stents, which is important in this high-risk population [14]. The main findings of our study suggest that the implantation of STENTYS self-expandable stents is safe and effective in the treatment of SVGs. No significant differences were found in angiographic and clinical parameters, such as late lumen loss, MACE and TVF between STENTYS BMS and STENTYS PES during the follow-up period.

A recent meta-analysis was performed comparing BMS versus DES in SVG lesions [8]. This meta-analysis compared the results of four other randomised controlled trials (RCTs): the ISAR-CABG [15], SOS; [16, 17], BASKET [18] and RRISC [19, 20]. The characteristics of these four trials are summarised in Tab. 4. Some differences between the ADEPT trial and the other RCTs are noteworthy. First, the number of patients included in the ADEPT is relatively small compared with the ISAR-CABG with 610 participants, although other trials show a similar population size [15]. The BASKET trial [18], with 47 participants, is the only trial that enrolled less patients than ADEPT. The other two RCTs enrolled 80 [16, 17] and 75 [19, 20] participants, respectively. A notable baseline difference is the mean age of the treated SVGs. In the ADEPT trial, the mean age of the graft was 16.2 years, which seems significantly older than in the other studies, where the age of the graft varies between 11 and 13.8 years [8]. Other baseline characteristics, such as cardiac risk factors, are similar to the other RCTs. The 12-month follow-up time of the ADEPT trial is the same as the ISAR-CABG trial, although shorter than the other three RCTs, which varied between 18 and 36 months. Also, less patients returned for follow-up angiography at 6 months compared with the other trials. This was despite multiple attempts by the study personnel to contact the patients.

**Table 4:** The characteristics of the four RCTs: ISAR-CABG, SOS, BASKET and RRISC

Trial	No of patients DES/BMS	TVR	Myocardial infarction	All-cause mortality	Type of DES	Follow-up
ISAR-CABG [15]	303/307	7%/13% *p* = 0.01	4%/6% *p* = 0.27	5%/5% *p* = 0.83	PES OR permanent SES OR biodegradable SES	12 months
SOS [16, 17]	41/39	15%/31% *p* = 0.08	15%/31% *p* = 0.10	12%/5% *p* = 0.27	PES	24 months
BASKET [18]	34/13	18%/46% *p* = 0.045	6%/0% *p* = 1.00	3%/15% *p* = 0.18	PES OR SES	18 months
RRISC [19, 20]	38/37	4.3%/24.5% *p* = 0.005	2.6%/0% *p* = 0.99	13.2%/29.7% *p* = 0.08	SES	6 months

*DES* drug-eluting stent, *BMS* bare metal stent, *TVR* target vessel revascularisation, *PES* paclitaxel-eluting stent, SES sirolimus-eluting stent

To place these data in the context of previous trials is challenging. When comparing late lumen loss at 6 months between the STENTYS self-expanding BMS (0.53 mm ± 1.09 mm) and STENTYS self-expanding PES (0.47 mm ± 0.95 mm), the data seem consistent with results in native coronary vessels and from similar studies of SVGs [8].

The paclitaxel-eluting version did not show improved angiographic follow-up results, while in the four historical randomised trials a difference in QCA outcomes in favour of DES was observed. This could be attributed to the limited sample size at baseline and the angiographic follow-up. Regarding to the clinical outcomes, the incidence of MACE at 6 months was similar in the ADEPT trial (14.0%) and the ISAR-CABG trial (10.7%), both due mostly to clinically driven TLR.

Finally, the high MACE rate found in in the literature and in this study confirms that SVGs are still different from native coronaries, and may require a different procedural approach.

## Limitations of the study

This study was underpowered to test the study hypothesis, because of a slower than anticipated enrolment. Therefore 57 patients were included in the study instead of 80, as planned. The lumen loss in the BMS group was higher than estimated in the statistical design: a larger number of patients than estimated may have been needed. However, the findings of this study remain concordant with similar studies. Larger randomised studies comparing self-expanding stents with balloon-expanding stents are needed to further determine differences and optimise clinical practice. Furthermore, this study was conducted with the older generation of paclitaxel-eluting STENTYS self-apposing stents. Late catch-up and thrombosis of paclitaxel-eluting stents have been reported. In the meantime, the current generation of the STENTYS self-apposing stent (STENTYS Xposition) has been modified to elute sirolimus. Also, optimal positioning was challenging with the older generation STENTYS stent because of shortening of the stent at deployment due to retracting an outer sheath which releases the stent from the distal edge. A novel balloon delivery system for the STENTYS SES, (the STENTYS Xposition), was therefore developed to improve accurate stent positioning[21].

## Conclusion

Treatment of SVGs with STENTYS self-expandable stents was safe, without showing any difference in outcomes between BMS vs PES arms. No significant differences were found in late lumen loss and MACE between STENTYS BMS and STENTYS PES during the follow-up period. Further larger randomised studies are needed to investigate differences between the two groups.
